# IL-7 and IL-8 inhibit gamete interaction in the zona-free hamster egg sperm penetration assay

**DOI:** 10.1155/S0962935192000139

**Published:** 1992

**Authors:** H. Lambert, I. Collazo, A. Steinleitner

**Affiliations:** 1 Department of Obstetrics and Gynecology Mount Sinai Medical Center Miami Beach FL USA; 2 San Francisco Reproductive Medicine Center 390 Laurel Street Suite 100 San Francisco CA 94118 USA

## Abstract

Current thought in reproductive endocrinology suggests hat endometriosis-associated subfertility may be the result of an adverse influence of activated immunocompetent cells on fertilization and embryo development. Inflammatory ediators such as interleukin-1 and tumour necrosis actor have been implicated in the pathophysiology of this process. The purpose of this study was to assess the effect of two recently characterized cytokines, interleukin-7 (IL-7 ) and interleukin-8 (IL-8), on gamete interaction in the perm penetration assay (SPA). Donor sperm were preincubated or 4 h with 0.5, 5, 50, or 500 ng ml^-1^ of human ecombinant IL-7 or IL-8. Sperm penetration was determined by an experienced gametologist by the presence of decondensed sperm heads or pronuclei formation. A dose-dependent inhibition of gamete interaction was observed following coincubation with either IL-7 or IL-8. These data offer the possibility that IL-7 and IL-8 may play a role in the pathogenesis of immunocompetent cell-associated subfertility.


Mediators of Inflammation 1, 67–69 (1992)

### IL-7 and IL-8 inhibit gamete interaction in the zona-free hamster egg sperm penetration assay

H. Lambert*, I. Collazo and A. Steinleitner[CA,*]

Department of Obstetrics and Gynecology, Mount Sinai Medical Center, Miami Beach, FL, USA
*Present Address: San Francisco Reproductive Medicine Center, 390 Laurel Street, Suite 100, San Francisco, CA, 94118, USA

[CA] Corresponding Author

**CURRENT thought in reproductive endocrinology suggests that endometriosis-associated subfertility may be the result of an adverse influence of activated immunocompetent cells on fertilization and embryo development. Inflammatory mediators such as interleukin-1 and tumour necrosis factor have been implicated in the pathophysiology of this process. The purpose of this study was to assess the effect of two recently characterized cytokines, interleukin-7 (IL-7) and interleukin-8 (IL-8), on gamete interaction in the sperm penetration assay (SPA). Donor sperm were preincubated for 4 h with 0.5, 5, 50, or 500 ng ml$^{-1}$ of human recombinant IL-7 or IL-8. Sperm penetration was determined by an experienced gametologist by the presence of decondensed sperm heads or pronuclei formation. A dose-dependent inhibition of gamete interaction was observed following coincubation with either IL-7 or IL-8. These data offer the possibility that IL-7 and IL-8 may play a role in the pathogenesis of immunocompetent cell-associated subfertility.**

**Key words:** Endometriosis, Infertility, Interleukins

### Introduction

Research over the last decade has led to a blurring of the distinctions between endocrinology, immunology, and reproduction. Inflammatory mediators which were presumed to function solely as regulators of immunocompetent cell function have been found to have wide ranging activities as growth factors and paracrine regulators of organ function.[1] Monokines and lymphokines have been shown to modulate ovarian steroidogenesis *in vitro* and have been hypothesized to contribute to various aspects of ovarian and endometrial homeostasis.[1,2] Activated peritoneal exudative cells have been demonstrated to inhibit early reproductive events in a rodent model.[3] These data have been offered in support of a hypothesis suggesting that alterations of the peritoneal inflammatory environment are responsible for impaired fertility in patients suffering from endometriosis and unexplained infertility.[4]

In this report we describe experiments characterizing the effect of interleukin-7 (IL-7, pre-B-cell growth factor) and interleukin-8 (IL-8, macrophage-derived chemotactic factor) on gamete interaction in the hamster egg sperm penetration assay (SPA).

### Materials and Methods

*Sperm preparation:* Semen specimens were obtained on five separate occasions from proven donors serving as fertile controls for the SPA performed in our clinical laboratory. Semen samples were allowed to liquify for 30–60 min at 37°C. Concentration and motility were assessed using a Makler chamber. The sample was divided into 0.5 ml aliquots and layered with 3.0 ml of Biggers, Whitten, Whittingham (BWW) media for overnight incubation.

*Sperm penetration assay:* Sexually mature female golden hamsters (6–8 weeks old) were subjected to ovarian hyperstimulation with intraperitoneal (i.p.) injections of 40 IU of pregnant mare's serum gonadotropins (PMSG, Sigma Chemical Company, St Louis, MO) on cycle day one followed by 40 IU i.p. of human chorionic gonadotropins (hCG, Sigma) 52–56 h later. Sixteen hours after hCG administration, hamsters were sacrificed by cervical dislocation for oocyte collection. The cumulus cell mass was dissipated with 0.1% hyaluronidase (Sigma). Zonae pellucidae were removed by treatment with 0.1% trypsin (Sigma). Eggs were rinsed twice and transferred to wells containing sperm suspensions. Sperm samples were adjusted to a final concentration of $1 \times 10^6$ spermatozoa/ml under the following conditions: neat BWW media, IL-7 or IL-8 at concentrations of 0.5, 5, 50, and 500 ng ml$^{-1}$. Following a 4 h incubation, oocytes were mounted and read. Penetration of hamster eggs was determined by counting swollen sperm heads and associated tails in cytoplasm.

*Cytokines:* Recombinant human IL-7 was provided by the Immunex Corporation, Seattle, WA. Human recombinate IL-8 was provided as a gift by Dr.

© 1992 Rapid Communications of Oxford Ltd.

**Table 1.** Effect of IL-7 and IL-8 on gamete interaction in the sperm penetration assay

| | | % penetration | | | |
|---|---|---|---|---|---|
| | neat BWW | 0.5 ng ml$^{-1}$ | 5 ng ml$^{-1}$ | 50 ng ml$^{-1}$ | 500 ng ml$^{-1}$ |
| IL-7 | 88.0 (44/50) | 53.8 (14/26) | 24.2[a] (8/33) | 0[a] (0/33) | 0[a] (0/24) |
| IL-8 | | 34.2[a] (12/35) | 12.7[a] (6/47) | 0[a] (0/44) | 0[a] (0/43) |

[a] $p < 0.001$, Kruskal–Wallis test

Kouji Matsushima of the National Cancer Institute.

*Statistical analysis:* Nonparametric analysis of variance (Kruskal–Wallis test) was used to evaluate the data. All values are reported as the mean ± the standard error of the mean.

### Results

Spermatoza preincubated in neat BWW (standard assay conditions) penetrated 44 of 50 hamster oocytes (88%). This result was consistent with the usual donor control in our laboratory which repeatedly achieved equal to or greater than 80% penetration. Preincubation with IL-7 and IL-8 produced a dose-dependent inhibition of fertilization (Table 1). Addition of either cytokine at concentrations of 5 and 50 ng ml$^{-1}$ completely inhibited gamete interaction.

In higher doses of IL-8 (50–500 ng ml$^{-1}$), vacuole formation characteristic of oocyte activation was observed in the cytoplasm of some eggs. These eggs were never found to have products of fertilization (i.e. swollen sperm head or pronuclei with accompanying mid-piece and tail) in their cytoplasm.

Longitudinal evaluation of sperm motion parameters demonstrated no acute effect of IL-7 or IL-8 incubation on sperm motility during the incubation period of the SPA (data not shown). With long term (24 h) exposure to IL-8 sperm motility dropped to approximately 20% of control values.

### Discussion

Contemporary thought suggests that infertility in patients with stage I/II endometriosis and some cases of unexplained infertility may be the result of an adverse influence of activated immunocompetent cells on early reproductive events.[4] This hypothesis is supported by several lines of experimental evidence. Peritoneal fluid from patients with endometriosis contains increased numbers of lymphocytes and activated macrophages as well as elevated levels of certain inflammatory mediators.[4–6] *In vitro* and *in vivo* gamete interaction is inhibited in the presence of peritoneal fluid obtained from endometriosis patients.[7,8] Studies by Hill and coworkers have demonstrated certain inflammatory peptides to have detrimental effects on measures of early reproductive performance *in vitro*. In their study, gamete interaction in the sperm penetration assay was markedly inhibited by gamma interferon and tumour necrosis factor in physiological concentrations.[9] Human granulocyte macrophage colony stimulating factor (GM-CSF), B-cell growth factor (BCGF), and interleukin-2 (IL-2) had no effect on gamete interaction or were inhibitory only in the highest concentrations tested. *In vitro* mouse embryo development was inhibited by gamma interferon, colony stimulating factor, and BCGF.[10] IL-2 had no effect on mouse embryo development; tumour necrosis factor and IL-1 were embryo toxic only in high concentrations.

Although aberrant humoral and cellular immune function have been implicated in the pathogenesis of infertility in endometriosis patients, the identity of the pathological effector(s) has not been adequately elucidated. While levels of peritoneal fluid inflammatory mediators (e.g., interleukin-1 (IL-1), tumour necrosis factor (TNF), and complement factor-3 (C3)) are more frequently elevated in endometriosis patients as compared to fertile controls and patients suffering from chronic pelvic adhesive disease, 40–60% of endometriosis patients test negative for the cytokine of interest in most reports.[5,6,11] This observation has led investigators to study additional humoral and/or cellular factors as contributors to the infertility observed in these patients.

The data obtained in this study permit us to add IL-7 and IL-8 to the list of inflammatory mediators known to exert a deleterious influence on gamete performance. Preincubation with as little as 0.5 ng ml$^{-1}$ of IL-7 or IL-8 produced a dramatic inhibition of penetration in the SPA. The increased incidence of oocyte activation observed suggests that the reduced penetration rate is the result of adverse influence on both sperm function and egg metabolism.

Although these data are provocative, as with most inflammatory mediators, it is difficult to assess the relevance of IL-7 and IL-8-induced inhibition of gamete interaction to the pathogenesis of immunocompetent cell-mediated subfertility. The dynamics of most inflammatory peptides (including IL-7 and IL-8) in peritoneal fluid and the reproductive tract have not been elucidated. At present IL-8 is thought to act primarily as a chemoattractant and activator of macrophages to sites of inflammation.[12] The physiological role of IL-7 is less clear. IL-7 is known to stimulate the proliferation of bone marrow-derived pre-B-cells[13] and to serve as a growth factor for T-cells.[14] There are no data describing tumour cell lysis or modulation of nonimmunocompetent cell metabolism by IL-8. The data obtained in this study offer the possibility that IL-7 and/or IL-8 may play a role in the pathogenesis of endometriosis-associated subfertility and unexplained infertility in these individuals. Additional studies characterizing the presence of IL-7 and IL-8 in peritoneal fluid and its relationship to the inhibition of *in vitro* gamete interaction and other inflammatory mediators such as TNF and IL-1 are in progress.

### References

1. Adashi E. The potential relevance of cytokines to ovarian physiology: the emerging role of resident ovarian cells of the white blood cells series. *Endocrin Rev* 1990; **11:** 454–464.
2. Tabibzadeh S. Evidence of T-cell activation and potential cytokine action in human endometrium. *J Clin Endocrinol Metab* 1990; **71:** 645–649.
3. Steinleitner A, Lambert H, Lauredo I. Heterologous transplantation of activated murine peritoneal macrophages inhibits gamete interaction *in vivo*: a paradigm for endometriosis-associated subfertility. *Fertil Steril* 1990; **54:** 725–729.
4. Halme J, Becker S, Haskill S. Altered maturation and function of peritoneal macrophages: possible role in pathogenesis of endometriosis. *Am J Obstet Gynecol* 1987; **156:** 783–789.
5. Fakih H, Baggett B, Holtz G, *et al.* Interleukin-1: possible role in the infertility associated with endometriosis. *Fertil Steril* 1987; **47:** 213–217.
6. Eisermann JE, Gast M, Pineda J, *et al.* Tumor necrosis factor in peritoneal fluid of women undergoing laparoscopic surgery. *Fertil Steril* 1988; **50:** 573–579.
7. Sueldo CE, Lambert H, Steinleitner A, *et al.* The effect of peritoneal fluid from patients with endometriosis on murine sperm-ova interaction. *Fertil Steril* 1987; **48:** 697–701.
8. Steinleitner A, Lambert H, Kazensky C, *et al.* Peritoneal fluid from endometriosis patients affects reproductive outcome in an *in vivo* model. *Fertil Steril* 1990; **53:** 926–929.
9. Hill J, Cohen J, Anderson D. The effects of lymphokines and monokines on human sperm fertilizing ability in the zona-free hamster egg penetration assay. *Am J Obstet Gynecol* 1989; **160:** 1154–1159.
10. Hill J, Haimovici F, Anderson D. Products of activated lymphocytes and macrophages inhibit mouse embryo development *in vitro*. *J Immunol* 1987; **139:** 2250–2254.
11. Halme J. Release of tumor necrosis factor-alpha by human peritoneal macrophages *in vivo* and *in vitro*. *Am J Obstet Gynecol* 1989; **161:** 1718–1725.
12. Baggiolini M, Walz A, Kunkel S. Neutrophil-activating peptide-1/interleukin-8, a novel cytokine that activates neutrophils. *J Clin Invest* 1989; **84:** 1045–1049.
13. Namen A, Lupton S, Hjerrild K, *et al.* Stimulation of B-cell progenitors by cloned murine interleukin-7. *Nature* 1988; **333:** 571–573.
14. Grabstein K, Namen A, Shenebeck K, *et al.* Regulation of T cell proliferation by IL-7. *J Immunol* 1990; **144:** 3015–3020.

ACKNOWLEDGEMENT. Supported by the Reproductive Biology Research Foundation, Torrance, CA, USA.

NOTE: Presented to the 38th annual meeting of the Society for Gynecologic Investigation, San Antonio, TX, March 20–23, 1991

**Received 21 October 1991;
accepted in revised form 18 November 1991**
